# Predictors of renal recovery in critically ill patients with AKI: observations from the ongoing FBI clinical trial

**DOI:** 10.1186/cc14378

**Published:** 2015-03-16

**Authors:** S Robinson, U Larsen, A Zincuk, S Zwisler, P Toft

**Affiliations:** 1 Odense University Hospital, Odense, Denmark

## Introduction

The predictive value of NGAL for renal recovery is not established.

## Methods

Data from the first 19 patients were assessed during a multicentre low molecular weight heparin trial (FBI, EudraCT Number: 2012-004368-23). Critically ill patients with AKI are randomly assigned into either a treatment arm (1 mg/kg enoxaparin) or a control arm (40 mg enoxaparin) upon commencement of CRRT. The primary outcome is the occurrence of venous thromboembolism. NGAL was measured at baseline and during CRRT-free intervals.

## Results

**Figure 1:**
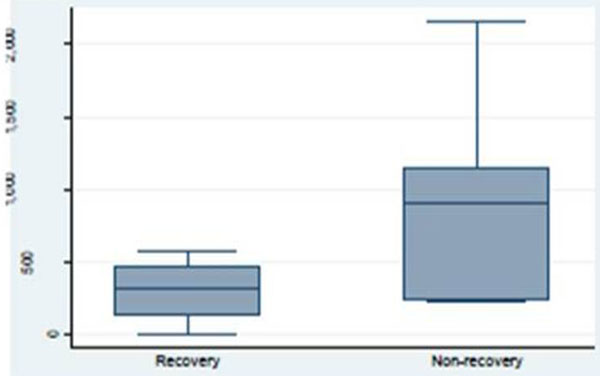
**Plasma neutrophil gelatinase-associated lipocalin concentration by recovery**.

**Figure 2:**
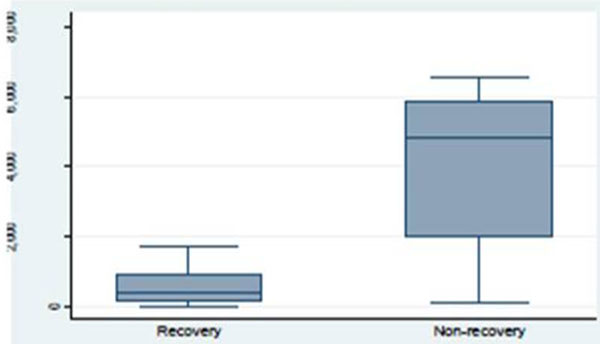
**Urine neutrophil gelatinase-associated lipocalin concentration by recovery**.

Patients were comparable at baseline with respects to demographics, APACHE II, creatinine, NGAL, start of dialysis, and the duration of dialysis. The main cause of AKI was sepsis (42%). In 63% of the patients, the reason for starting dialysis was a combination of anuria and electrolyte disturbances. Twenty-six percent of patients were dialysis dependent after the first CRRT-free interval. Plasma NGAL levels were higher in nonrenal recovery patients (1,074 (±694) ng/ml) compared with renal recovery patients (296 (±197) ng/ml; *P *= 0.01). Urine NGAL levels were higher in nonrenal recovery patients (3,885 (±2,722) ng/ml) compared with renal recovery patients (597 (±565) ng/ ml; *P *= 0.006). See Figures 1 and 2. Inflammatory parameters (WBCs, CRP, and procalcitonin) did not differ significantly between the groups.

## Conclusion

NGAL may be able to predict renal recovery, and allow proper utilization of resources.

